# Building permits—control of type IV pilus assembly by PilB and its cofactors

**DOI:** 10.1128/jb.00359-24

**Published:** 2024-11-07

**Authors:** Nathan A. Roberge, Lori L. Burrows

**Affiliations:** 1 Department of Biochemistry and Biomedical Sciences, and the Michael G. DeGroote Institute for Infectious Disease Research, McMaster University, Hamilton, Ontario, Canada; Geisel School of Medicine at Dartmouth, Hanover, New Hampshire, USA

**Keywords:** type IV pilus assembly, motor ATPases, regulation, secondary messengers, phage-encoded inhibitors

## Abstract

Many bacteria produce type IV pili (T4P), surfaced-exposed protein filaments that enable cells to interact with their environment and transition from planktonic to surface-adapted states. T4P are dynamic, undergoing rapid cycles of filament extension and retraction facilitated by a complex protein nanomachine powered by cytoplasmic motor ATPases. Dedicated assembly motors drive the extension of the pilus fiber into the extracellular space, but like any machine, this process is tightly organized. These motors are coordinated by various ligands and binding partners, which control or optimize their functional associations with T4P machinery before cells commit to the crucial first step of building a pilus. This review focuses on the molecular mechanisms that regulate T4P extension motor function. We discuss secondary messenger-dependent transcriptional or post-translational regulation acting both directly on the motor and through protein effectors. We also discuss the recent discoveries of naturally occurring extension inhibitors as well as alternative mechanisms of pilus assembly and motor-dependent signaling pathways. Given that T4P are important virulence factors for many bacterial pathogens, studying these motor regulatory systems will provide new insights into T4P-dependent physiology and efficient strategies to disable them.

## INTRODUCTION

Type IV pili (T4P) are surfaced-exposed protein filaments found in many species of bacteria and archaea (1, 2). T4P are members of a functionally diverse superfamily of structurally related nanomachines that include type II secretion systems (T2SS), which export folded proteins and protein complexes; DNA uptake systems; and archaeal pilus or flagellar systems (1). These filaments undergo dynamic cycles of extension and retraction (3–5) enabling bacteria to sense surface contact (4, 6–8), interact with their environment, including adhering to other cells (9–11), take up large biomolecules such as DNA (12–14) or bacteriophages (15, 16), and to move along surfaces (13, 17, 18). These appendages often serve as virulence factors, facilitating infection and colonization of host tissues (10, 11, 19). Despite the broad functional diversity of various T4P systems, the ability to assemble and extend a pilus fiber is an essential first step (17, 20). Pilus extension relies on one or more (14) homohexameric ATPases and organisms that lack an extension ATPase are unable to assemble surface-exposed pili (17, 21, 22). Here we will discuss the T4aP system as a model, as it is the best-studied T4P subfamily (1).

**Fig 1:**
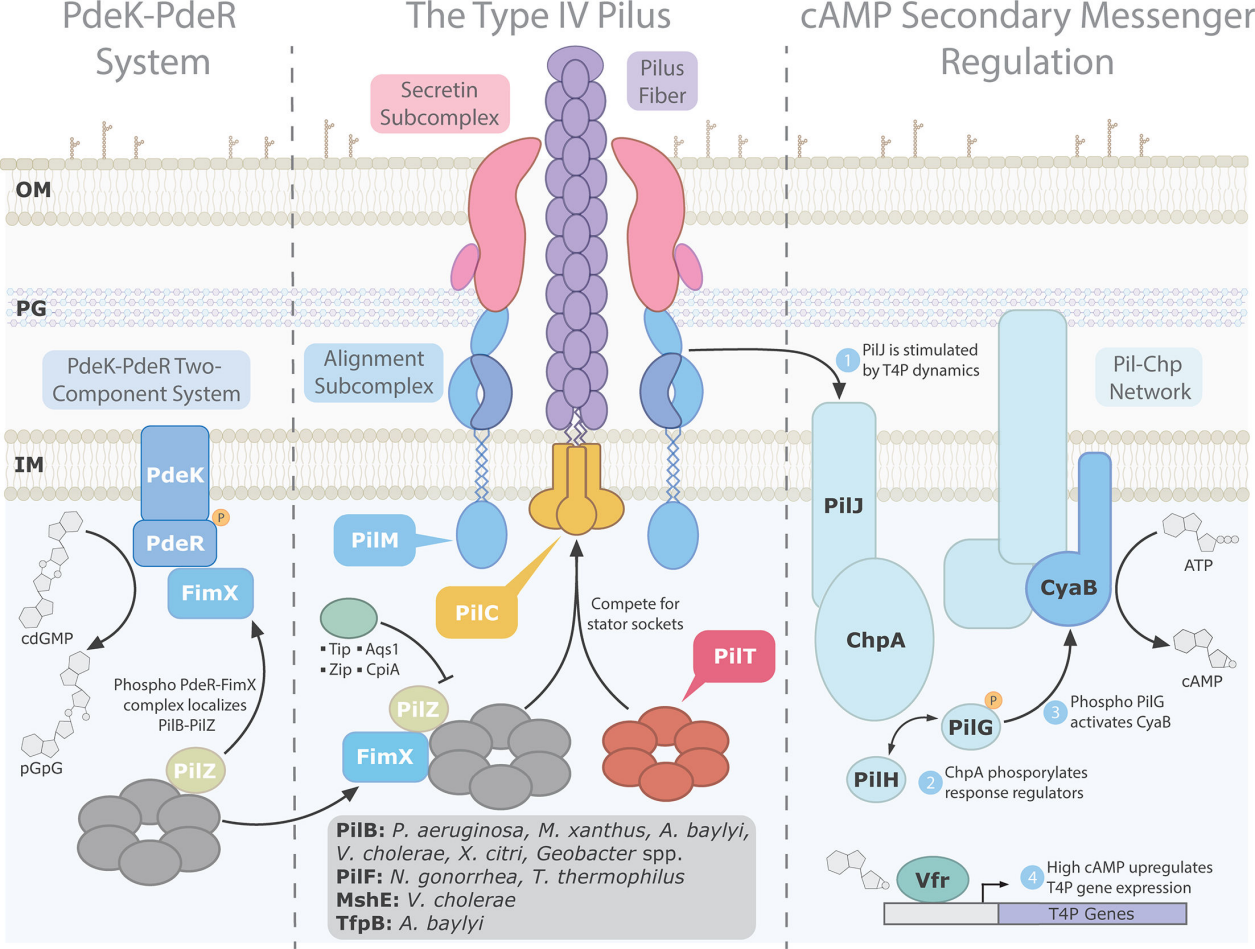
The type IV pilus (T4P) and various regulatory systems. The type IV pilus (center) is a large protein nanomachine that spans the width of the cell envelope. It comprises four structural subcomplexes: the pilus fiber, alignment, secretin, and cytoplasmic motor subcomplexes. For simplicity, not all structural components are shown or labeled. Cytoplasmic extension motor ATPases, potentially in complex with protein effectors (FimX or PilZ), interface with the platform protein (PilC) within the stator socket (PilM) at the base of the pilus. ATP hydrolysis by extension motor ATPases constrained by the stator supply energy to the platform protein, driving pilin polymerization and extending the pilus fiber. Extension and retraction (PilT) ATPases transiently compete for stator binding. Phage- (Tip, Aqs1, and Zip) or host-encoded (CpiA) inhibitory peptides bind to PilB or the PilZ-PilB complex (Zip) to disable pilus extension. The Pil-Chp chemotaxis network (right) generates cyclic-AMP (cAMP) to positively regulate the expression of T4P components. Inner membrane methyl-accepting chemotaxis protein PilJ receives an undefined signal transmitted through pilus dynamics. This triggers phosphorylation of the complexed histidine kinase ChpA, which transfers the phosphoryl group to response regulators PilG and PilH. Phosphorylated PilG stimulates the activity of adenylate cyclase CyaB, which converts ATP to cAMP. cAMP is bound by the transcription factor Vfr to upregulate the transcription of T4P and other genes. The PdeK-PdeR two-component system (left) is required for proper PilB and FimX subcellular localization in certain species. An unknown signal stimulates sensor kinase PdeK, triggering the phosphorylation of the response regulator PdeR. PdeR contains an EAL domain which is activated upon phosphorylation to degrade local pools of cyclic-di-GMP (cdGMP). FimX affinity for PdeR increases at low cdGMP concentrations, forming a complex to properly localize PilB-PilZ to T4P machines.

T4aP filament assembly in well-studied model organisms such as *Pseudomonas aeruginosa* and *Myxococcus xanthus* requires the ATPase PilB, which docks in a cytoplasmic socket formed by the stator protein PilM at the base of the pilus machine (Fig. 1) (23, 24). PilB interfaces with the integral membrane platform protein, PilC (22, 23, 25, 26). Recent *in silico* studies of PilC and a T2SS homolog PulF from *Klebsiella pneumoniae* strongly implicate the formation of a functional trimer, where the two cytoplasmic domains of each PilC monomer interface symmetrically with the six protomers of the ATPase hexamer (27, 28). ATP hydrolysis-powered conformational changes constrained by PilM are transmitted to PilC and interacting major pilin (PilA) subunits. PilA subunits are ultimately extracted from their reservoir in the cytoplasmic membrane (26, 29) and polymerized to form a pilus fiber (30, 31), though the details of PilC’s role remain unknown (32). The pilus is extruded through the periplasm, guided by the cylindrical alignment subcomplex, and finally through a gated secretin pore in the outer membrane (25, 33, 34). Extended pili are depolymerized during retraction through the action of a separate antagonistic ATPase, PilT, that engages with PilC within the same cytoplasmic socket (17, 35), returning PilA subunits to the inner membrane for subsequent rounds of assembly. In *P. aeruginosa*, PilB and PilT exchange are predicted to occur through stochastic competition, where both ATPases transiently compete for empty T4P machinery (20). Although the role of retraction ATPases in transducing signals related to surface contact has been the focus of prior work (36), this review highlights the control of pilus assembly, the first step before retraction.

**Fig 2:**
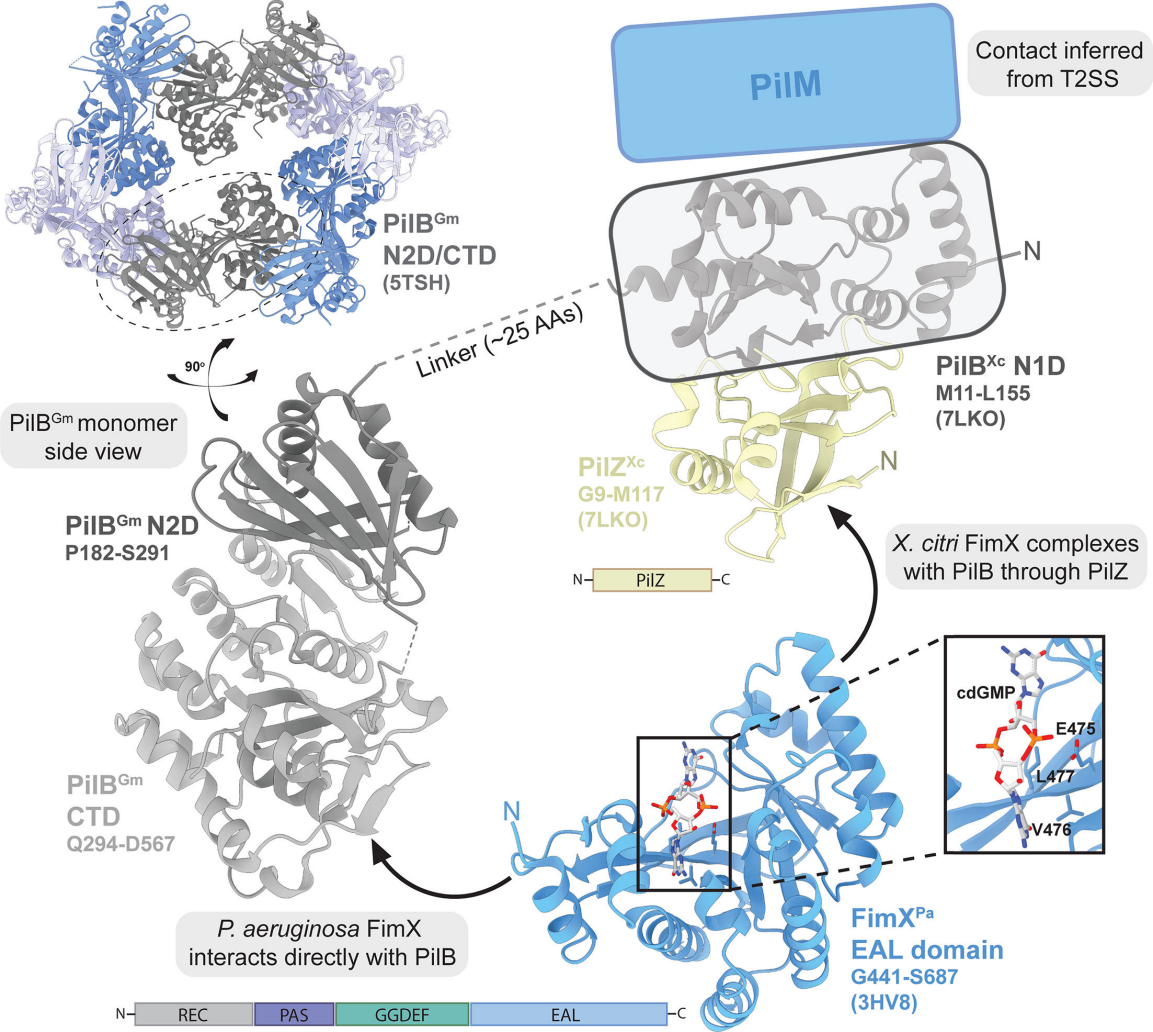
PilB, PilZ, and FimX interactions and associations. The PilB hexamer displaying C2 symmetry derived from the X-ray crystal structure of *Geobacter metallireducens* (PilB^Gm^, 5TSH). The PilB CTD and N2D (gray) side profile are shown. The N1D equivalent is derived from the crystal structure of *X. citri* (Xc) N1D domain (gray) in complex with PilZ (beige, 7LKO). PilZ domain architecture is shown. The crystal structure of the *P. aeruginosa* (Pa) FimX EAL domain monomer (blue, 3HV8) bound to cyclic-di-GMP (cdGMP), and the full-length protein domain architecture is also shown. Inset highlights the FimX cdGMP-binding site and critical residues implicated in ligand interaction are indicated. Arrows indicate discrepancies in FimX association mechanisms, either directly binding to PilB (*P. aeruginosa*) or ternary complex formation with PilZ-PilB (*X. citri*). PilB N1D domain contacts with PilM are inferred from crystal structure interactions observed for their T2SS homologs (4PHT and 2BH1).

Extension ATPases such as PilB belong to the PilT-like family of the Additional Strand Catalytic “E” superfamily (29, 37). They have three domains, and all have a catalytic glutamate to mediate ATP hydrolysis, in addition to Walker A and Walker B motifs essential for substrate coordination (29, 38, 39). Each monomeric subunit adopts a bi-lobed tertiary structure comprised of an N-terminal domain (N2D) connected to a C-terminal domain (CTD) by a flexible linker region (Fig. 2). Assembly ATPases contain an additional general secretory pathway (GSPII) domain at their N-terminus (N1D) which is less well characterized due its flexibility, so far evading attempts to define its structure (29, 38, 39). This domain likely interfaces with the stator complex (40, 41), as similar interactions have been observed for ATPase homolog in the T2SS (42–44). These enzymes also have a conserved zinc coordination motif in the CTD, which is essential for function (29, 45).

X-ray crystal structures of PilB orthologs from *Geobacter* spp. and *Thermus thermophilus* bound to non-hydrolyzable ATP analogs revealed that these enzymes assemble into toroidal homo-hexamers, with the N2D of one protomer resting upon the CTD of the adjacent protomer (29, 38, 39). The ring has distinct topological faces, with differences in charge distribution and amino acid conservation across homologs (29). The residues at the interior of the toroid are more conserved while those at the perimeter and base are less conserved. Interestingly, rather than sixfold symmetry, these enzymes consistently exhibit elongated twofold symmetry (29, 38, 39). This oblong symmetry results from differential ligand occupancy of the six active sites, situated at the protomer-protomer interfaces. Four sites adopt a “closed” nucleotide-bound conformation while two remain in the “open” state, primed to bind available ATP. The binding cleft at the protomer interface cooperatively influences surrounding subunits upon substrate binding and hydrolysis (29). ATP hydrolysis therefore occurs directionally, with adjoining PilB subunits exerting differential conformational forces upon the next subunit in the chain. These distortions propagate around the torus and may, in turn, drive PilC rotation and translation in the membrane.

Given that pilus assembly is critical to the function of T4P, it is interesting that many bacteria and phages have evolved mechanisms to modulate the activity of these enzymes. PilB thus serves as a checkpoint for input from various secondary messenger molecules and protein effectors, which collectively permit the building of a pilus. While some of these regulatory strategies are widespread in T4P-expressing bacteria, others are unique to certain organisms, which begs the question, how are T4P dynamics regulated at the level of pilus extension?

## PILB REGULATION BY SECOND MESSENGERS

### cAMP and the Pil-Chp system

In specific bacteria, the expression of key components of the T4P machine, including the motor proteins, is positively regulated through increases in levels of cyclic-AMP (cAMP) (46, 47). This secondary messenger signaling is controlled through the Pil-Chp chemotaxis system (Fig. 1), homologous to the flagellar Che-cluster (48, 49). In the current *P. aeruginosa* model, methyl-accepting chemotactic receptor PilJ receives an as-yet unidentified signal. The stimulus is potentially associated with tension exerted upon retracting pili (7, 50), surface-stiffness-induced PilA diffusion dynamics in the inner membrane (51), and/or molecular inputs from cytoplasmic retraction machineries (52). This signal is transduced to the histidine kinase ChpA (7). ChpA activation results in the phosphorylation of response regulators PilG, which activates the adenylate cyclase CyaB (7, 49), and PilH, potentially serving as a phosphate sink (53). CyaB activation increases intracellular levels of cAMP, which upregulates transcription of T4P-associated genes when bound to virulence factor regulator (Vfr), a homolog of the catabolite repressor protein, Crp (47).

In rod-shaped bacteria like *M. xanthus* or *P. aeruginosa*, T4P are assembled at the cell poles (34, 54–56). Pilus function therefore necessitates recruitment of the essential cytoplasmic ATPases to the machines at the poles. Asymmetric recruitment of extension and retraction ATPases is thought to determine the direction of twitching motility, with polar switching events causing reversal (22, 56–59), though it is worth noting that alternative expression techniques influence the polar distribution of fluorescently tagged motor proteins leading to some conflicting reports (22, 54, 57, 58). Recent studies suggest that PilG and PilH directly influence T4P ATPase localization (58, 59). Fluorescently tagged PilG and PilH have biased subcellular localization patterns, with PilG primarily accumulating toward the leading pole of individually twitching *P. aeruginosa* cells and PilH displaying a more diffuse distribution (59). Biased PilG polar recruitment is hypothesized to positively upregulate T4P assembly at the leading pole through increased signaling by the Pil-Chp network mechanosensory relay (58). Asymmetric polar recruitment of fluorescently tagged PilG and PilB is correlated, with the PilB signal diffusing throughout the cytoplasm in a Δ*pilG* background. Deletion of *pilH*, however, serves to further increase the asymmetric polarization of PilB in cells. Coupled with the inability of *pilH* mutants to reverse twitching direction upon collisions with other cells, the data suggest a model where PilG promotes PilB polar recruitment at the leading pole driving twitching motility, while PilH action serves to counteract this positive feedback loop (58, 59). Despite this evidence that PilG and PilH influence PilB localization in *P. aeruginosa*, to date no interactions between these proteins have been reported. The question therefore remains as to the mechanism of PilB localization and whether other modulators facilitate PilG-dependent recruitment.

### cdGMP and the PilB N-terminal domain

Cyclic dimeric-GMP (cdGMP) is a ubiquitous secondary messenger involved in coordinating bacterial lifecycle transitions by modulating metabolism, various forms of motility, production of extracellular polymeric substances, and biofilm formation (60). T4P are among the cellular components responsive to cdGMP-dependent regulation. The controlled synthesis or breakdown of this molecule catalyzed through the action of diguanylate cyclases (DGCs) and cdGMP phosphodiesterases (PDEs), respectively (60), in local subcellular pools is thought to modulate pilus assembly or function in response to environmental cues (61). For example, in *M. xanthus,* the loss of function of only three of 24 predicted cdGMP metabolizing enzymes decreased T4P-dependent motility (62), highlighting their specificity. There are a variety of mechanisms by which cdGMP modulates pilus activity. T4P-dependent aggregation in *Clostridioides difficile* is regulated through a cdGMP-binding riboswitch upstream of the primary pilus operon that increases its expression at high secondary messenger concentrations (63). The action of T4P has also been linked to maintenance of cdGMP homeostasis. In *P. aeruginosa*, the DGC SadC interfaces with PilO of the T4aP alignment subcomplex, which, in turn, modulates cdGMP catalysis in response to surface contact (8, 64).

Some T4P extension ATPases are regulated through direct binding of cdGMP (65–67). The presence of an additional GSPII domain at the N-terminus (N1D) distinguishes T4P extension ATPases from retraction ATPases (67, 68). The role of these domains in the broader context of pilus extension is poorly understood due to their poor resolution in available structures (29, 38, 39). The T4P-associated extension ATPases of certain bacterial species can bind cdGMP at the N1D (66, 67, 69, 70), a requisite for proper function (66, 69, 71). This ability was initially described for the *Vibrio cholerae* protein MshE, from the mannose-sensitive hemagglutinin (Msh) pilus system.

The N-terminal domain, called MshEN, binds to cdGMP *via* two 24 amino acid motifs linked by a five amino acid spacer. These atypical motifs differ considerably from other well-characterized cdGMP-binding sequences but are widely distributed among bacterial species (19, 65, 70, 72). The two motifs coordinate both guanine bases of the ligand through extensive side-chain hydrophobic interactions, notably three conserved leucine residues which form triangles around the nucleotides to stabilize them (69). Mutation of various residues within the motif attenuated Msh-dependent phenotypes, further supporting the hypothesis that cdGMP binding to MshE is essential for pilus function (65, 69). Altering two of three conserved leucine residues, L10A and L58A, significantly impacted motility and surface pilus production. Unexpectedly, however, mutation of all three conserved leucine residues to alanine abolished cdGMP binding *in vitro* but when that variant was used to complement a *mshE* mutant, restored the deficient phenotypes, suggesting that this version of the enzyme is constitutively active (69). A follow-up study in *V. cholerae* showed that retraction dynamics of the Msh pilus were mediated by the cdGMP-bound functional state of MshE. While pilus retraction itself is governed by a separate ATPase, PilT, modulating the intracellular levels of cdGMP directly influenced surface pilus production. Deletion of four DGCs significantly reduced detectable surface pili while upregulating flagellar motility. Notably, none of the DGCs interacted with MshE in a bacterial 2-hybrid (BACTH) assay, suggesting instead that global intracellular cdGMP levels modulated function. Msh pilus retraction dynamics are therefore thought to be contingent upon the degree of MshE-dependent extension, which is prioritized at higher cdGMP concentrations and fine-tuned by nucleotide availability (71).

Other bacteria harbor conserved cdGMP-binding extension machinery and may adopt similar strategies to promote pilus assembly. The PilB2 extension ATPase ortholog from *Clostridium perfringens* encodes a cdGMP-binding motif in its N-terminal domain that is nearly identical to that of *V. cholerae* MshEN. PilB2 is capable of high-affinity allosteric cdGMP binding *in vitro*, though titration of the secondary messenger did not influence ATP hydrolysis rates (72), hinting at a potentially more complex regulatory mechanism than simply modulating activity.

PilB in the predatory bacterium *M. xanthus* was also recently shown to bind cdGMP *via* an N-terminal MshEN domain (70). Mutations to the nucleotide-binding motif impacted T4P-dependent S-motility, surface piliation, and biofilm formation, consistent with phenotypic observations in *V. cholerae* (66, 71). Many of the mutants exhibiting the most significant reductions in T4P-dependent phenotypes had decreased levels of PilB. Thermal stability of purified His-tagged PilB MshEN domain was increased in the presence of cdGMP, supporting a mechanism of nucleotide-dependent stabilization of the domain. However, a G6L substitution in this domain significantly reduced intracellular PilB levels while simultaneously increasing S-motility to levels greater than the wild type. Generation of a *M. xanthus* PilB mutant equivalent to the triple leucine substitution which rendered MshE from *V. cholerae* constitutively active failed to complement S-motility and abolished PilB expression, indicating inconsistencies between model organisms (70). Taken together, this suggests a multifaceted mechanism of enzymatic regulation or hints at additional species-specific regulatory modes.

The functional contribution of the MshEN/N1D to catalysis and extension is poorly understood, though the recent cryo-EM structural characterization of full-length ATPases could provide more clues. The N1D from the *T. thermophilus* PilB homolog PilF revealed a distinct two-subdomain ring configuration when the MshEN domain was docked into the resulting density (68). When comparing the APO and AMPPNP bound states of the full-length protein, the N1D undergoes a downward and outward displacement, accompanied by an outward expansion of the N2D and CTD. The authors hypothesized that the N1D engages with additional components of the inner membrane T4P platform, such as the stator ring, to drive the assembly of the pilus fiber in response to ATP hydrolysis. In this model, the ATP-powered movement of the N1D could potentially provide the mechanical energy necessary to drive pilus assembly (68). In a separate study, PilF binding to cdGMP was confirmed through NMR spectroscopy (67), perhaps pointing to a mechanism where cdGMP binding influences N1D-dependent engagement with the other ATPase domains or proteins of the pilus apparatus. Indeed, *in vitro* cdGMP binding at the N1D of full-length PilB from *Chloracidobacterium thermophilum* selectively reduced the binding affinity of ADP but not ATPγS (73), suggesting that conformational perturbations in the N-terminal domain could be communicated to the catalytic domains to modulate nucleotide selectivity and function.

## PROTEIN EFFECTORS THAT MODULATE PILB FUNCTION

### PilZ and FimX

PilB localization and function are subject to additional post-translational regulation by various binding partners (Fig. 2). In multiple species, PilZ and FimX interface with PilB to modulate its function (16, 41, 74–76). PilZ proteins are small (~120 AAs) and although it belongs to the “PilZ” superfamily of cdGMP binding proteins, PilZ itself does not bind this signaling molecule (41, 74). Nevertheless, PilZ knockouts of *P. aeruginosa*, *Xanthomonas citri*, and *Lysobacter enzymogenes* lack surface piliation and twitching motility (16, 41, 77). In *X. citri*, structural studies revealed that PilZ binds residues 12–163 of PilB, corresponding to the N1D (29, 38, 39). Co-expression of PilZ and PilB in a recombinant system drastically improved the solubility of the latter, suggesting a role in aqueous stabilization (41). This result is consistent with NMR HSQC spectral data indicating that the flexibility of the PilB N1D was reduced upon the addition of PilZ (41). Thus, PilZ may act as a stabilization factor for the disordered PilB N1D in some species.

A separate regulator, FimX, is also necessary for proper pilus assembly and twitching motility in a variety of organisms (41, 75, 76, 78). Fluorescently tagged PilB polar localization is reduced in Δ*fimX* strains, indicating that this protein could be necessary for trafficking the motor ATPase to the pilus machinery (41, 76). FimX mutation has also been implicated in altering the polar distribution of fluorescently tagged PilT (76), potentially hinting at a more intricate regulatory mechanism supporting both extension and retraction systems. Alternatively, at elevated levels of cdGMP, FimX is dispensable for pilus formation and twitching motility in *P. aeruginosa* (79), perhaps suggesting a role in maintaining T4P function over broad cellular concentrations of this secondary messenger. In contrast to PilZ, FimX is a large protein (~700 AAs) containing REC, PAS, GGDEF, and EAL domains. CheY-like REC domains generally act as receptors—accepting a phosphoryl-transfer from a cognate kinase—though the FimX REC domain lacks the conserved aspartate residue needed to catalyze this transfer (80). Despite this, deletions of the REC domain in *P. aeruginosa* were reported to result in mis-localization both of FimX and pilus assembly, with extended filaments observed along the lateral side of the cell body (80). These data, which need more detailed verification of pilus machinery mis-localization, indicate that FimX could play a role in restricting the polar assembly of the pilus machine. PAS domains often serve as molecular sensors of environmental or metabolic changes, but the factor(s) that might be sensed by this domain for FimX is unknown (81).

The canonical functions of the GGDEF and EAL domains are the synthesis and hydrolysis of cdGMP, respectively (60, 65, 82). Interestingly, the GGDEF domain of FimX is degenerate, though the EAL domain retains limited cdGMP binding and weak hydrolysis activity (80, 83, 84). In FimX, the degenerate GGDEF motif (GDSIF) lacks DGC activity but is predicted to retain a weak affinity for GTP binding (75, 83). Mutating the GDSIF motif to a non-GTP-binding AASIF motif in *P. aeruginosa* results in defective surface piliation and twitching motility. The addition of GTP increased the *in vitro* PDE activity of wild-type FimX but not of the AASIF mutant (61), indicating that GTP binding by the GDSIF motif modulates cdGMP binding or hydrolysis in the adjacent EAL domain, a mechanism observed in other dual domain “hybrid” enzymes (85). The capacity for cdGMP binding is required for FimX-PilB interaction in *P. aeruginosa*, as truncation of the EAL domain or mutation of the catalytic triad to AAA were sufficient to abolish interaction in split luciferase and size exclusion co-elution assays. Under these conditions, full-length FimX eluted at a molecular weight consistent with homodimerization (76). However, whether the EAL domain participates in dimerization contacts is less clear. An X-ray crystal structure of monomeric *P. aeruginosa* FimX EAL domain bound to cdGMP revealed significant clashes when superimposed with the dimeric GGDEF-EAL APO state structure. In a small-angle X-ray scattering envelope reconstruction, additional lobes of density between dimeric full-length and ΔEAL domain FimX were observed at opposite ends of the complex, away from the dimerization interface (83). The crystal structure of a dimeric *P. aeruginosa* FimX EAL domain has also been solved, but it was bound to the cdGMP hydrolysis product pGpG (84), suggesting that substrate hydrolysis could modulate EAL-specific association or metabolite-induced conformational rearrangements.

In *Xanthomonas oryzae*, the impact of FimX on pilus extension is subject to an additional layer of regulation through interactions with the PdeK-PdeR two-component system (Fig. 1) (78). The response regulator PdeR has an active EAL domain with PDE activity. FimX polar localization was abolished in mutants lacking either component or when phosphorylation-dependent PDE activity of PdeR was disabled. Pull-down experiments indicated the formation of a ternary complex, and microscale thermophoresis studies led to the conclusion that FimX interacted with both proteins, but that affinity for PdeR was reduced at elevated levels of cdGMP. This suggested a model where pilus function is impaired at elevated levels of cdGMP due to weak FimX-PdeR interaction. Following stimulation by an unknown signal, PdeK phosphorylates PdeR, degrading cdGMP in the local area and enhancing interactions with FimX, forming a ternary complex with PdeK (78). This complex may, in turn, recruit PilB-PilZ to facilitate pilus extension.

Species-specific FimX coordination may not be limited to *Xanthomonas*, however. In *P. aeruginosa*, FimX is encoded with FimW, a separate cdGMP-binding extension regulator required for optimal surface piliation and twitching motility (86). Fluorescently tagged FimW accumulates rapidly at the cell poles in a cdGMP-dependent manner upon surface contact, suggesting that it too plays a role in surface sensing or in coordinating the assembly machinery. While FimW contributes to the regulation of extension dynamics, the exact function of this protein remains unknown and there is as of yet no evidence of any functional interaction with FimX or PilB.

Direct PilZ and FimX interactions have also been widely reported. Complexes of the two proteins from *Xanthomonas* spp. have been crystallized (41, 87, 88), providing evidence that PilB-PilZ-FimX ternary complexes are biologically relevant. This three-way interaction has been demonstrated *in vitro*, where the addition of the FimX GGDEF-EAL domains to a pre-formed *X. citri* PilZ-PilB N1D complex shifted the size-exclusion chromatogram elution profile of the total complex. Furthermore, the PilB-PilZ-FimX ternary complex hydrolyzed over twice the units of ATP *in vitro* compared to PilB-PilZ alone (41). Curiously, however, the addition of exogenous cdGMP did not influence ATPase activity under the experimental conditions tested (41), in contrast to the reported binding requirements of PilB-FimX in *P. aeruginosa* (76). This is not the only reported discrepancy in PilZ-FimX interactions between *P. aeruginosa* and *Xanthomonas* spp. orthologs. Isothermal titration calorimetry studies previously failed to demonstrate an interaction between *P. aeruginosa* PilZ and the FimX EAL domain (89). Indeed, the critical residues for PilZ contact, W445, M487, M488, and A493 in *X. citri* FimX, are not conserved in the *P. aeruginosa* ortholog (89), supporting the observed functional divergence. Together, this could suggest alternative binding modes or ternary complex organization between PilB-PilZ-FimX from various species.

### CpiA involved in DNA uptake

Natural transformation is the process by which cells take up DNA from the environment (90) and for many bacteria, T4 competence pili are essential for this process (14, 90). A recent study leveraged the natural transformation capacity of *Acinetobacter baylyi* by conducting a transposon-insertion screen to select genes contributing to pilus function (14). These genes were identified through loss of the ability to take up various DNA substrates. Among the expected hits in T4P structural genes was an uncharacterized transcriptional regulator, *cpiR,* whose loss resulted in a 100-fold reduction in transformation efficiency. The *cpiR* gene is encoded adjacent to a second uncharacterized gene, *cpiA*. Expression of *cpiA* under a separate inducible promoter was sufficient to reduce transformation, indicating CpiA transcription was likely suppressed through the action of CpiR. Notably, *A. baylyi* encodes two phylogenetically distinct extension ATPase homologs, PilB and TfpB, both of which independently contribute to pilus extension dynamics. Deletion of *cpiR* in a Δ*pilB* background caused no change in piliation, whereas deletion of the repressor in a Δ*tfpB* background abolished surface piliation. Coimmunoprecipitation experiments with GFP-tagged CpiA and PilB further confirmed an interaction between the two proteins, indicating that CpiA is a selective inhibitor of PilB but not TfpB (14).

While it is evident that *A. baylyi* encodes an additional regulator to modulate pilus function, questions remain as to the mechanism. The regulatory implications of selectively inhibiting only PilB are not well understood. Furthermore, the inhibitory mechanism of CpiA binding to PilB remains unclear, though understanding these molecular features could point to distinct structural or functional discrepancies between ATPases. Similar questions surrounding the functional origins of CpiA proteins in bacteria are also worth considering. CpiA is not encoded by other sequenced strains of *Acinetobacter*, leading the authors to speculate that CpiA was perhaps acquired through prophage incorporation (14), similar to other reported instances of naturally occurring PilB inhibitors (15, 91).

## PHAGE-ENCODED PEPTIDES THAT REGULATE PILB LOCALIZATION AND FUNCTION

For many bacteria, a consequence of having surface-exposed T4P is susceptibility to certain bacteriophages (15, 92, 93). Phages are viruses that specifically infect bacterial cells, and the first step of their infection cycle involves recognizing and binding to some type of accessible receptor on the cell surface (92). T4P are commonly used as primary phage receptors as they facilitate the translocation of viral particles toward the cell body upon retraction (92, 93). Some lysogenic phages that integrate into the host chromosome as a replication strategy have evolved mechanisms to disable T4P function in the form of peptide inhibitors. These are expressed by the prophage and prevent further infection of the same cell by related phages, a phenomenon termed superinfection exclusion (15, 91, 94, 95).

Recently, multiple members from a family of phage-encoded peptides were independently characterized and found to inhibit pilus formation and twitching motility by targeting the extension motor ATPase in *P. aeruginosa* (15, 91). The first of these, the twitching inhibitory protein (Tip) is encoded by the *P. aeruginosa-*specific lysogenic phage D3112. Tip binds selectively to PilB and not the retraction ATPase PilT. Expression of Tip alone delocalized GFP-PilB from the cell poles and attenuated twitching motility (91). BACTH assays with full-length Tip and PilB truncations starting from the N-terminus revealed a putative binding region between residues 187 and 227, corresponding to the PilB N2D. Subsequent mutagenesis studies implicated PilB residues P208 and Y209 as critical for Tip interaction. Furthermore, expression of PilB variants with point mutations of those residues in a Δ*pilB* background failed to rescue twitching motility, indicating that they are essential for PilB function (91). It remains unclear, however, whether Tip binding was eliminated due to improper PilB folding or oligomerization rather than by loss of the Tip binding interface. Similar mutations have drastically altered the function of PilB. For example, in a separate study, researchers screened for phages from hospital wastewater capable of lysing clinical isolates of *P. aeruginosa*. One phage-resistant mutant that arose during propagation had a PilB T278P substitution, which significantly reduced twitching motility (96). This residue also maps to the N2D and is in close proximity to P208/Y209, indicating that this region of PilB is critical for function.

**Fig 3:**
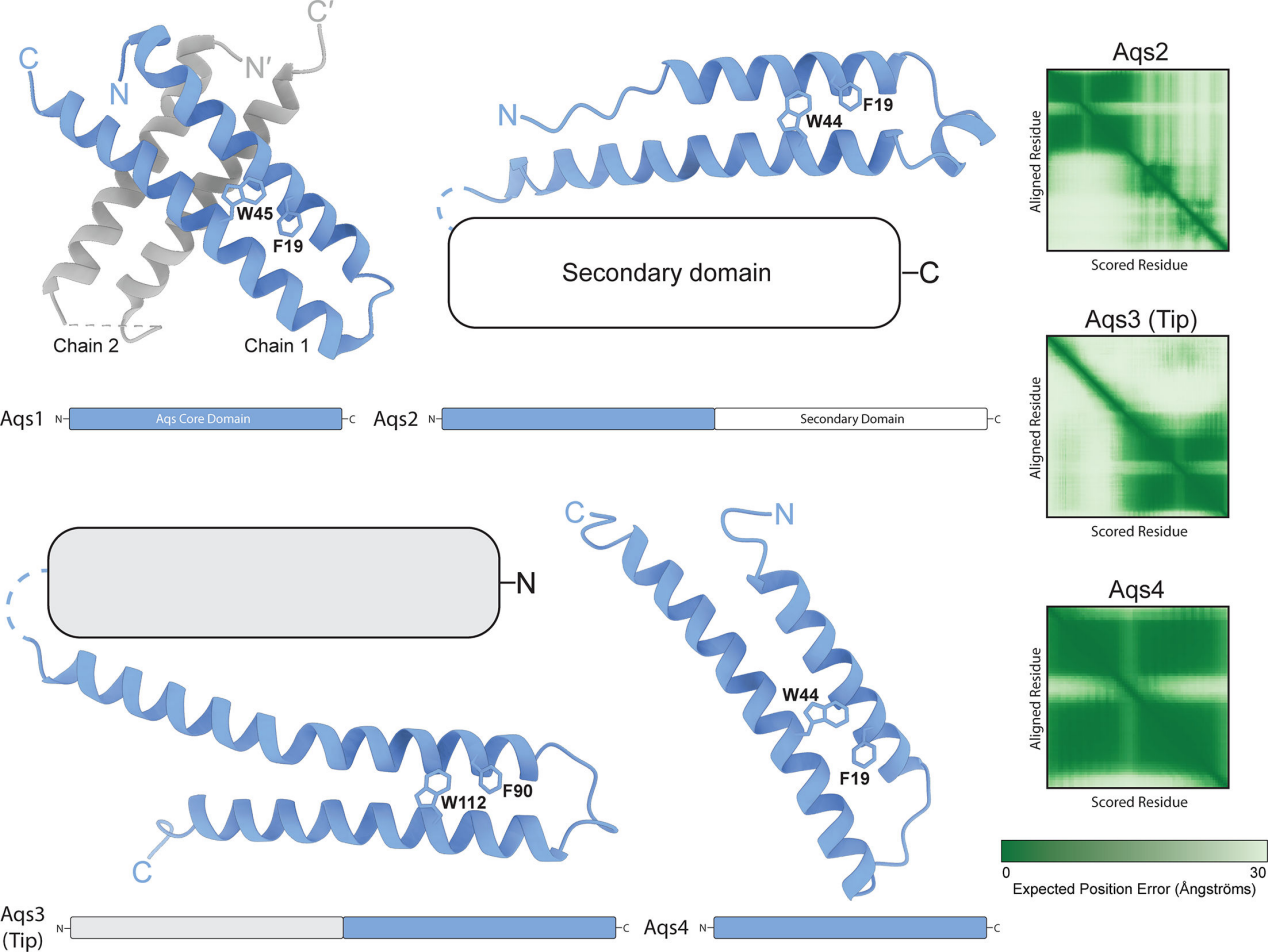
Phage-encoded PilB inhibitor peptide homologs. The X-ray crystal structure of the anti-quorum sensing 1 (Aqs1) protein dimer (6V7U, **M1-A63**) and Alphafold3-derived models of homologs Aqs2-4 encoded by other phages. The Alphafold3 predicted aligned error (PAE) plots denoting the estimated positional error for each residue (darker green being a more confident prediction and lighter green being less confident) are shown to the right. Critical residues for Aqs1 interaction with PilB, F19, and W45, are conserved in the other homologs. Aqs2 and 3 each have unrelated secondary domains of unknown function. The Alphafold3 models for Aqs2, 3, and 4 are available in ModelArchive (www.modelarchive.org) with the accession codes ma-c8zt4, ma-y35xz, and ma-vwby6, respectively.

Another T4P-targeting peptide from *P. aeruginosa* phage DMS3 was recently described. Remarkably, this peptide, anti-quorum sensing protein 1 (Aqs1), displayed dual binding to both the quorum sensing master regulator LasR (97) and PilB (15). Expression of Aqs1 inhibited twitching motility and conferred resistance to pilus-specific phages. BACTH assays using site-specific point mutants showed that F19 and W45 of Aqs1 were critical for PilB interaction. Aqs1 was crystallized as a dimer, revealing a helix-turn-helix motif with the predicted PilB-binding faces oriented outwards from the 4-helix bundle (Fig. 3). When crystallized bound to LasR, only one protomer of Aqs1 contacted the target, suggesting that the second protomer scaffolds the engaged monomer. Bioinformatic analyses identified homologs of putative PilB-inhibitory peptides from other related phages. Notably, phage-encoded peptides Aqs2 and Aq3, from JBD24 and JBD26, respectively, are modular and contain a secondary domain, in addition to the conserved PilB-binding core region (Fig. 3). Aqs3 is identical to Tip, though encoded by a different phage (91). These secondary domains are poorly modeled by Alphafold3 (plDDT <50), making it difficult to ascribe putative function. They could perhaps serve as structural components to stabilize the conserved Aqs core interface should these proteins remain monomeric in solution. Alternatively, the secondary module could provide the capacity to bind other targets, like Aqs1 with LasR. Regardless, the core domains of each protein share high sequence identity including the essential F and W residues found in Aqs1 (15). Intriguingly, a truncated form of Tip (Aqs3) lacking both putative PilB binding residues identified for Aqs1 was recently shown to retain some PilB interaction and attenuate *P. aeruginosa* infection of *Drosophila* (98). This result strongly suggests the contribution of other as-yet uncharacterized residues to PilB binding. Furthermore, the exact inhibitory mechanism of Aqs1 or Tip has yet to be clarified, including whether these two proteins even share a common PilB inhibition strategy. Given that Tip expression caused delocalization of GFP-PilB from the cell poles (91), phage-encoded peptides might disrupt hexamerization of the ATPase or prevent functional association of PilB with other T4P components such as PilC or PilM. Nevertheless, more studies are required to probe the structural basis of phage-encoded peptide binding and inhibition of PilB.

Phage-mediated control of T4P extension dynamics does not occur only through binding to PilB. In a separate study, a phage-encoded PilZ-binding peptide that indirectly modulates the function of PilB was identified. The PilZ interacting protein (Zip), from *P. aeruginosa* phage JBD26, disabled T4P function and conferred phage resistance when expressed from a plasmid. Notably, however, Zip expression in its native context from the lysogen did not abolish surface pilus production but rather significantly reduced the average pilus length, as quantified using fluorescent cysteine maleimide labeling of pilins. Zip deletion from the prophage caused a significant reduction in phage titers, indicating that this protein was responsible for carefully balancing pilus function to maintain virion populations (99). Lysogenic phages frequently encode repressor elements that provide immunity to infection by genetically similar phages (99, 100). The authors hypothesized that the shorter pili produced by lysogens expressing Zip reduced the number of phage progeny infection events. This “anti-Kronos” effect protects phage progeny from self-recognition and infection of established bacterial lysogens, resulting in genomic silencing (99). This strategy of tuning PilB function through control of PilZ binding therefore maintains functional T4P dynamics while preventing the ineffective deployment of viable phage particles. While certainly an exciting finding, several questions remain as to the nature of the mechanism. For example, how does Zip interface with PilZ and PilB, and what is the structural basis for altering function? Answering these questions could help inform on the nature of PilZ chaperone function and perhaps shed some light on PilZ-N1D functional interactions with the pilus machine.

## WHAT IS THE PURPOSE OF HAVING MULTIPLE PILB HOMOLOGS?

Some bacteria encode multiple T4P extension ATPases (14, 71). For certain organisms, this accommodates multiple functionally distinct nanomachines (71, 101). Extension ATPases from one system are generally incompatible with the other machineries, such as in the case where extension of *V. cholerae* Msh and competence (Com) pili requires the specific actions of MshE or PilB, respectively (101). One study illustrated this functional specificity by ectopic expression of various ATPase orthologs from *Pseudomonas stutzeri*, *Escherichia coli* (102), or *A. baylyi* in *V. cholerae* backgrounds that were extension- or retraction-deficient, assessing the frequency of Com pilus-dependent natural transformation. While the retraction ATPases displayed a degree of promiscuity and could complement function in the heterologous species, non-native extension ATPases failed to restore transformation, highlighting the additional specificity unique to the assembly ATPases. Heterologous PilB orthologs were unable to interact with *V. cholerae* PilC in a BACTH assay, suggesting that the enzymes may fail to interface correctly with the platform to drive extension. In addition, full-length sequence alignments of PilB homologs across species showed that the N1Ds, which are predicted to interact with the stator complex (40, 41), were the most diverse regions (103). Together, this could indicate that extension ATPase engagement with the structural components at the base of the pilus is highly species-specific, supporting the lack of promiscuous complementation.

For some bacteria, multiple ATPases are needed to coordinate extension in a single system. *A. baylyi* encodes two phylogenetically distinct ATPases, PilB, and TfpB (14). Retraction-deficient mutants with individual deletions of PilB or TfpB still assembled pili, though the percentage of piliated cells was significantly reduced in the TfpB background while the PilB mutant had wild-type levels. A double ATPase mutant produced no detectable pili. The discrepancies between the individual ATPase mutants indicate that PilB and TfpB perform distinct functional roles in T4P extension (14). TfpB was hypothesized to aid in efficient priming of pilus assembly, and PilB to maintain extension processivity over time. Moreover, the two enzymes might form a biologically relevant complex to optimally modulate pilus extension. If so, could this complex comprise two discrete hexamers or is the formation of PilB-TfpB heterooligomers a possibility? Interactions between ATPases have been observed in BACTH assays between extension-extension and extension-retraction homologs from *V. cholerae* and *P. aeruginosa* (24, 101, 104). This may indicate that inter-ATPase or subunit functional associations are biologically significant, though it is worth noting that BACTH assays performed in *E. coli* lack the additional context of the pilus structural components and relevant regulatory protein effectors. Nevertheless, functional associations between separate T4P ATPases have been extensively documented in retraction mechanisms (36). Some bacteria encode multiple PilT homologs and/or conditionally essential secondary paralog ATPases (PilU), which function optimally when coupled with a primary retraction ATPase (21, 22, 50, 104–106). More investigations are required to determine how interactions between ATPases may regulate extension, and this remains an interesting avenue for further research. Putative TfpB homologs are predicted to be encoded by other bacteria as well (14), suggesting that other pilus systems may employ similarly complex mechanisms.

## ALTERNATIVE MECHANISMS OF PILUS EXTENSION

Many T4P systems modulate pilus dynamics through the competition of cognate extension and antagonistic retraction machineries (17, 20). In bacteria such as *P. aeruginosa* or *M. xanthus,* deletion of the primary extension ATPase results in no detectable surface pili, while deletion of the retraction machinery results in a hyper-piliated but non-motile phenotype, owing to an inability to disassemble the fibers (11, 17). For other bacteria, disruptions to putative retraction ATPases can result in a loss of detectable surface pili, suggesting that these enzymes play a dual role in extension and retraction dynamics. In *Francisella tularensis*, surface filaments were no longer detected using transmission electron microscopy when either the putative extension or retraction ATPases were individually mutated. Complementation restored piliation, indicating that both ATPases were somehow involved in modulating extension (107). More recently, the primary retraction ATPase PilT from *V. cholerae* was shown to be essential for the extension of the Msh pilus (71, 101). This process was retraction-specific but did not require ATP hydrolysis by PilT. Inactive Walker Box mutants of PilT were still capable of pilus retraction through cooperation with the auxiliary retraction ATPase PilU, and these cells displayed wild-type levels of surface piliation. Detectable pili were lost, however, when PilT was deleted or when PilT and PilU were both inactivated, confirming that retraction dynamics mediated through PilT were also required for efficient pilus assembly. Interestingly, various suppressor point mutations in the major pilin subunit MshA were sufficient to restore the piliation defect of the Δ*pilT* mutant (101). These data led to a proposed model where PilT-dependent retraction primes pilus assembly, after which processive extension can commence through MshE activity. The MshA suppressors are thought to bypass the need for retraction-dependent priming, perhaps by allowing for spontaneous disassembly of the pilus (108, 109), enabling MshE to act immediately.

Force-dependent spontaneous assembly of pili has also been observed in *Neisseria gonorrhoeae,* indicating that pilus extension is possible in the absence of extension systems. Optical trap microscopy experiments, which quantify the T4P-dependent deflection of a bead suspended in a laser trap, showed that at low levels of PilT, the application of sufficient forces to the beadinduced pilus elongation (110). Extension machineries, in cooperation with the platform protein, normally provide the energy necessary to overcome the activation threshold for extracting a pilin from the cytoplasmic membrane and incorporating it into the pilus, but external forces pulling on a pilus also facilitate pilin polymerization. Perhaps this is accomplished by decreasing the energy required to add pilins to the base of a fiber. These findings collectively support the idea that the natural elasticity of the pilus fiber (111) likely contributes to assembly and disassembly dynamics.

Some organisms lack separate retraction machinery, with a single bifunctional ATPase providing both pilus assembly and disassembly functions (1, 112, 113). These type 4c pili are the second largest subfamily in bacteria (114) and are thought to coordinate pilus dynamics by employing two distinct platform proteins to interface with one ATPase, instead of the single platform protein with multiple ATPases configuration found in type 4a systems (1, 113). The tight adherence (Tad) pilus from *Caulobacter crescentus* is a well-studied example (4, 114). In this organism, fluorescently tagged fusions of the ATPase CpaF localized to sites of both single pilus extension and retraction. Moreover, the rates of both extension and retraction were influenced when point mutations impacting ATP hydrolysis by CpaF were introduced, indicating that this enzyme was responsible for mediating both processes (114). While functional data strongly implicate these enzymes as performing multiple roles, the structural basis for this dual activity is less clear. Recent cryo-EM structures of CpaF bound to its nucleotide substrates provide evidence for the assembly of toroidal homo-hexamers (113). Consistent with structures of T4a motor proteins (29, 38, 39), CpaF rings also adopt C2 elongated symmetry. Cycling between an “open” state with high affinity for ATP, a substrate-bound catalytically active “closed” state, and nucleotide release “openʹ” state induces clockwise motion of the ATPase in keeping with right-handed pilus extension. As for retraction, the authors suggest that shifting the affinity for ATP between the open and openʹ states could induce conformational changes potentiating counterclockwise motion. An allosteric switch, potentially in the form of N-terminal domain engagement with the ATPase or post-translational modification, was proposed to trigger the change in active site affinities (113), but more studies are needed to identify such a signal.

## PILB AS A REGULATOR OF OTHER PROCESSES

T4P are deployed during early biofilm formation to facilitate initial surface attachment and microcolony formation (11, 86, 115). These processes typically rely on functional pilus dynamics for cells to make surface contact followed by stable adherence, triggering a cascade of surface-associated responses (86). For *M. xanthus*, however, the T4P extension ATPase PilB is involved further downstream in the regulation of exopolysaccharide (EPS) production, required for biofilm maturation. Mutants of *M. xanthus* that are unable to generate pili are also deficient in EPS production (116), but this pilus-dependent decrease is suppressed by mutations in the PilB active site. For example, an M388I mutation in the Walker B motif that did not impact T4P-dependent S-motility—indicating active ATP hydrolysis—restored EPS production when introduced in a background where the major pilin was deleted. Inactivation of the Walker A motif by a K327A mutation also upregulated EPS production but abolished S-motility. Together, these data indicate that some direct contribution of PilB, rather than the assembly of a pilus filament, is required for the suppression of EPS production (116). Purified *T. thermophilus* PilF with an equivalent Walker A mutation had decreased affinity for a fluorescently labeled ATP analog (116), suggesting that it is the substrate-free PilB state that mediates this signaling, though more thorough biochemical characterizations are still required. Perhaps allosteric regulation by cdGMP is partially responsible for directing PilB signaling events. For certain ATPases, *in vitro* studies showed that cdGMP binding within the N1D is independent of ATP binding or hydrolysis, though ligand binding does appear to influence nucleotide affinity for the PilB active site between protomers (67, 73). How a nucleotide-free PilB conformation influences N1D configuration or cdGMP access is unclear, but answering this question could potentially point toward a mechanism of secondary messenger sensing by the pilus machinery to coordinate other important phenotypes.

Direct PilB regulatory inputs have to date been reported only in *M. xanthus,* though other bacteria may respond similarly using various regulatory effectors. For example, *P. aeruginosa* FimX mutants display altered biofilm matrix composition and have impaired signaling in response to nitric oxide stimulation (117). In both instances, how these proteins mediate signaling events downstream of pilus extension is unknown. Mutations in PilB that impact pilus extension may also indirectly contribute to signaling outputs through other systems. In *P. aeruginosa*, the expression of PilA is positively regulated by the PilSR two-component system, and accumulation of PilA in the inner membrane downregulates this pathway. Transcriptomic analyses have implicated PilSR as important for the expression of flagellar structural and regulatory components, acting upstream of the FleSR transcriptional hierarchy (118). Loss of PilB—and thus pilus extension—that leads to accumulation of PilA in the cytoplasmic membrane, and the subsequent dampening of PilSR signaling could therefore have broader consequences for cellular physiology. As such, certain phenotypes attributed to the loss or mutation of PilB may be indirectly due to changes in the expression of downstream genes. More studies are needed to disentangle these regulatory pathways for a more complete understanding of PilB-dependent signaling.

## INHIBITING EXTENSION ATPASE FUNCTION AS AN ANTI-VIRULENCE STRATEGY

T4P are important virulence factors for many bacteria, facilitating initial attachment and invasion of host tissue (10, 11, 19, 119, 120). A better understanding of pilus extension mechanisms could inform the rational design of anti-virulence inhibitors targeting T4P. Extension ATPases have long been proposed as potential therapeutic targets to limit the virulence of priority pathogens (119, 121). Complications with targeting such components can arise, however, as small molecule inhibitors of ATPases often have unacceptable promiscuity, particularly if they are nucleotide-like (122).

Some attempts have been made to identify small molecule inhibitors of T4P-associated ATPases (119, 121, 123). In one recent study, a fluorescent nucleotide reporter assay was adapted for high-throughput screening using the robust *C. thermophilum* T4P motor ATPase. A pilot screen identified the compound quercetin as having ATPase inhibitory bioactivity, with subsequent attenuation of *M. xanthus* twitching motility and pilus assembly (123). Unfortunately, quercetin is a common pan-assay interference compound (124) that has also been identified as a mitochondrial ATPase inhibitor (125), illustrating the difficulties encountered in traditional small molecule drug discovery. In a follow-up study by the same group, the *in vitro* screen was modified to instead quantify free phosphate released from ATP hydrolysis. Screening over 2300 compounds identified two structurally related previously-approved drugs used to treat Parkinson’s disease, as competitive inhibitors. Treatment with either drug disrupted *M. xanthus* surface piliation and T4P-dependent motilities of both *M. xanthus* and *Acinetobacter nosocomialis* in a dose-dependent manner (121), emphasizing the therapeutic potential of these compounds.

Cell-based high-throughput screens have also yielded lead compounds capable of disabling pilus extension. A microscopy-based screen, quantifying the disruption of *Neisseria meningitidis* T4P-dependent microcolony formation on endothelial cells, identified 163 hits from a library of over 2,200 compounds. Three compounds, each with diverse chemical structures, were selected for further characterization. Treatment with these compounds disrupted *N. meningitidis* surface piliation and cellular aggregation, supporting the hypothesis that the pilus extension machinery was the target. Further supporting this idea, incubation with each of the compounds reduced the ATPase activity of the N-terminally truncated assembly motor PilF but had no effect on the retraction motor PilT, highlighting the mechanistic specificity (119).

In another recent study, molecular dynamics simulations combined with *in silico* docking of compounds from an approved drug library were used to identify molecules potentially capable of restricting the conformational changes induced by substrate binding within the PilB hexamer (126). This virtual screen revealed five lead compounds predicted to bind to key PilB catalytic residues. While certainly a good proof of concept, these compounds have yet to be biochemically assessed for PilB inhibition, which will ultimately determine the validity of this approach.

As a separate line of inquiry, the phage-encoded inhibitory peptides described above represent naturally occurring and highly specific templates for the design of inhibitors. While inhibition of T4P dynamics by many phage-encoded peptides occurs through binding to PilB, the precise nature of the inhibitory mechanism(s) and the relevant protein-protein interactions are less clear. Nevertheless, unpacking the molecular mechanisms of PilB inhibition by phage peptides provides an exciting opportunity for the development of potent T4P inhibitors based on designs that have been honed by millennia of co-evolution.

## CONCLUSIONS AND NEXT STEPS

The function of T4P extension ATPases is modulated by a variety of molecular inputs. It stands to reason that regulation of pilus extension rather than retraction would be favored. Control of retraction dynamics first requires the resource-intensive process of building a pilus, so regulatory pathways aimed at the level of extension benefit from restricting or tuning assembly only when pili are needed. In *P. aeruginosa*, PilT is predicted to dwell within the stator complex for longer durations than PilB (20), consistent with the idea that the pilus extension step is more sensitive to regulation. To this end, bacteria have adopted various regulatory strategies, some of which are widespread and conserved, while others are highly specialized.

Many open questions remain as to the nature of these regulatory discrepancies and their implications for bacterial physiology. For example, why do some extension machineries but not others bind directly to secondary messenger molecules? Similarly, why do certain extension systems require post-translational regulation by protein effectors? Perhaps the answer lies partially in the diversity of bacterial lifestyles which shapes pilus function. In *P. aeruginosa*, a single T4aP system is optimized for motility (115) but is also essential for responding to surface contact and promoting adherence (7, 58, 86, 115). For this organism, the ability to tune pilus dynamics and respond accordingly to physical or environmental signals is essential. This could reasonably be accomplished through input from various effectors or receptors that sense secondary messenger gradients to modulate efficiency, such as FimX binding to cdGMP (79). By contrast, an ATPase like *V. cholerae* MshE requires direct cdGMP binding for function and is therefore optimally deployed under high cdGMP sessile conditions to promote cellular aggregation (69, 71). The expression of multiple extension ATPases that act on the same pilus machine in *A. baylyi* may also reflect a strategy to modulate pilus assembly in response to the right combination of environmental or cellular cues (14). Furthermore, some bacteria appear to lack easily identifiable extension regulatory effectors. New evidence from *Geobacter sulfurreducens* suggests that T4P are necessary to secrete the subunits of polymeric cytochrome filaments that are involved in electron transfer to external substrates (127). Perhaps T4P function in those species is more akin to T2SS endopilus production (28, 128), and thus the lack of extension regulators is a functional adaptation to ensure a population of shorter pili for optimal secretion?

Several questions remain as to the stoichiometric arrangement of regulatory partners and their collective interactions with the remainder of the machine. How is PilB able to engage with the stator and platform proteins when complexed with various protein effectors? A model of the *X. citri* pilus machinery suggests that each PilB N1D domain is bound by a PilZ monomer, supported by size-exclusion chromatography data examining complex formation (41). It remains unclear, however, whether PilZ remains bound to PilB or if subunits disengage and then re-engage over the course of the ATP hydrolysis cycle. Where FimX fits into this model, and in what ratio, is also unclear. Does the PilB-PilZ-FimX ternary complex stay together once PilB engages with the stator or platform proteins, and if so, how is FimX configured? Characterizing these collective interactions will provide a more comprehensive understanding of the pilus extension process and assist in reconciling species-specific functional discrepancies.
